# Jacob F. Frantz, M. D.

**Published:** 1914-04-15

**Authors:** 


### OBITUARY.

Jacob F. Frantz, M. D.—Doctor Frantz, the President of the Dentists Supply Co., died at his home in New Rochelle, N. Y., February 7th, at the age of 62. Dr. Frantz was a graduate of medicine in 1876. In 1879 he organized the Wilmington Dental Manufacturing Co., for the manufacture of porcelain teeth. In 1895 he went to New York as president of the Consolidated Dental Manufacturing Co., and in 1899 organized the Dentists' Supply Co., which in fifteen years has become one of the largest manufacturers of porcelain teeth in the world. He was a good organizer and an indefatigable worker, and he has made a great success of the business which he more largely than any one else created. He was personally and agreeable gentleman and had lots of friends in the profession and among the trade people. He was a good citizen and held many offices of trust in his home town. His death seems untimely and he will be missed in the Dental trade world.

